# Business datasets and record linkage: Correlates of linkage and estimating risks of non-linkage biases

**DOI:** 10.23889/ijpds.v1i1.396

**Published:** 2017-04-19

**Authors:** Jamie Moore, Gabriele Durrant, Peter W. Smith

**Affiliations:** 1 Department of Social Statistics and Demography and ADRCE, University of Southampton

## Objectives

Our first objective is to investigate how between subject differences in the likelihood of record linkage consent and record linkability determine the composition of and so risks of biases in estimates from linkable business datasets. The utility of datasets linking information from multiple sources is compromised by such non-linkage biases, but both components of the linkage process have rarely been considered. Our second objective is to introduce methods for evaluating non-linkage bias risks in datasets. Such evaluations can inform linkage method choice and assessment of the validity of linked dataset findings. Previous work, often lacking non-linked subject information on non-sample dataset covariates, tends to utilise overall linkage rates as quality measures, but in the related area of survey non-response correlations between analogous response rates and non-response biases are weak.

## Approach

We utilise the UK 2010 Small Business Survey (SBS) dataset. If a survey subject consents to record linkage, an attempt is made to append their Inter-Departmental Business Register (IDBR) identifier (if one exists), enabling linkage to other surveys etc. Given this, we evaluate bias risks arising from variation in subject linkage consent and identifier appendability, as well as its product, overall linkability, utilising representativeness indicators developed to evaluate survey non-response bias risks. These measure risks in terms of sample-subset similarity (representativeness) given an attribute covariate set obtained from the sample dataset, based on variation in subject inclusion propensities estimated by logistic regression, and are decomposable to assess correlates of inclusion propensity variation. Specifically, we use the CV (the standard deviation of inclusion propensities divided their mean), computed given nine attribute covariates describing business demography and perceived performance.

## Results

We give full details in our presentation. Briefly, overall CVs suggest the linkable dataset exhibits substantial non-representativeness and non-linkage bias risk. Decompositions suggest main impacts on the linkable dataset are under-representation of very small businesses (those with low turnovers, few employees and / or un-incorporated), due to being both less likely to consent and less likely to have an identifier appended, and under-representation of businesses unable / refusing to respond to survey items, due to being less likely to consent.

## Conclusion

Our analyses provide evidence of non-linkage bias risks in linked SBS datasets caused by under-representation of several sample subgroups. Each is explicable given known IDBR under-coverage or knowledge of business response processes. We also conclude that representativeness indicators are an easily applied method by which such risks can be evaluated.

